# Authority reliance vs. deliberative assessment in processing online rumors: evidence from fNIRS

**DOI:** 10.3389/fnhum.2026.1750499

**Published:** 2026-02-24

**Authors:** Shuhua Cheng, Xinyue Yang, Yi Ding

**Affiliations:** 1 School of Business Management, Zhejiang Financial College, Hangzhou, China; 2 School of Business, Anhui Xinhua University, Hefei, China; 3 School of Economics and Management, Anhui Polytechnic University, Wuhu, China

**Keywords:** fNIRS, online rumors, source credibility, tagging warnings, third-party fact-checkers

## Abstract

**Purpose:**

This study aimed to clarify how the authority of a fact-checker shapes neurocognitive processing of online rumors. Specifically, this study examined differences in neural responses to corrections provided by authoritative and non-authoritative sources.

**Approach:**

Functional near-infrared spectroscopy (fNIRS) was used to measure neural activity in the prefrontal cortex while participants evaluated information that had been fact-checked by either authoritative or non-authoritative third-party sources. Behavioral metrics, such as judgment accuracy, were collected alongside neural data to correlate brain activity with decision-making outcomes.

**Results/findings:**

Authoritative fact-checkers produced stronger activation in the left prefrontal cortex (LPFC) and improved overall judgment accuracy, suggesting a cognitive “fast track” that facilitates information acceptance. This enhanced accuracy was accompanied by increased LPFC engagement, indicating deeper analytical engagement. For true information, non-authoritative fact-checking led to reduced right prefrontal cortex (RPFC) activation and only marginal behavioral improvements, suggesting participants relied on heuristic shortcuts or “cognitive offloading” rather than rigorous deliberation. During false information processing, RPFC activation decreased across specific channels (e.g., Ch19), with non-authoritative sources yielding higher false-information judgment accuracy (59%) compared to authoritative sources (55%). This paradoxical effect suggests that lower source credibility can, in certain contexts, elicit more vigilant evaluation of false claims. The neural and behavioral responses to authoritative versus non-authoritative sources varied based on information veracity, consistent with cognitive dissonance theory, which posits adaptive shifts in processing strategies in response to credibility cues.

**Value:**

By linking source credibility to distinct neural signatures and accuracy outcomes, this work provides a neurocognitive account of how fact-checker authority influences belief updating. The findings highlight that credibility cues can promote heuristic acceptance or more careful analysis, depending on the situation. Furthermore, this evidence can inform more effective rumor-intervention strategies that are sensitive to both source attributes and information type.

## Introduction

The rapid proliferation of online rumors has emerged as a pressing concern in academia and various fields (Pal et al., 2019; Pennycook et al., 2020). Rumors are defined as information and news lacking confirmation or certainty regarding factual accuracy (Bondiell and Marcelloni, 2019). Sharing messages of unknown veracity may cause panic and anxiety among the public, especially when they eventually turn out to be false (Li and Sakamoto, 2014). The proliferation of online information has imposed a constant responsibility to determine its credibility. Notably, individuals tend to rely on the perceived authority of a source, demonstrating higher trust in the information if it originates from an expert or official institution (Moran and Nechushtai, 2022; Tandoc et al., 2018; Yang et al., 2026; Kaňková and Matthes, 2026; Bolsen and Druckman, 2015). While efficient, this heuristic can lead to passive acceptance without deeper thought (Kaňková and Matthes, 2026). Understanding the cognitive and neural basis of this process, especially when people evaluate corrective information from sources of varying credibility, is crucial for addressing the spread of rumors.

Prior research found that the use of denials is an effective strategy to debunk rumors (Pal et al., 2019; Bordia et al., 2005). Adding warning labels has proven useful in enhancing individuals’ critical thinking ability, thereby curtailing the spread of rumors (Pal et al., 2019; Pennycook et al., 2020). Bolsen and Druckman (2015) revealed that warnings are more effective than corrections in countering directional reasoning regarding scientific claims. Furthermore, Pennycook et al. reported that warning labels can significantly improve accuracy in identifying political information (Pennycook et al., 2020). Notably, the impact of the authority of third-party fact-checkers on information dissemination and public perception remains poorly understood.

Across platforms such as social media, news reports, online forums, and expert blogs, fact-checking efforts exert inconsistent effects on users’ beliefs (Bhattacherjee, 2023). Factors such as the authority and professionalism of third-party fact-checkers significantly shape users’ cognitive frameworks and information processing strategies to varying degrees (Xu et al., 2023). Prike et al. (2024) found that authoritative labels serve as effective interventions, reducing the spread of false information and enhancing users’ ability to discern true from false information. For instance, official media outlets, due to their authority and credibility, tend to have their messages more readily accepted and internalized as personal beliefs by users despite containing imperfections. This process is intricately linked to psychological mechanisms such as trust transfer and cognitive biases (Feng et al., 2023; Moore et al., 2021). In contrast, social media information is more fragmented and diverse, necessitating users to sift through and assess authenticity and reliability on their own, which increases cognitive load and may lead to but also risks information overload and misjudgment (Qiang and Hu, 2025). Thus, investigating how the authority of third-party fact-checkers influences individual cognitive processing holds significance for understanding decision-making behaviors of information recipients.

The authority of an information source depends on the public’s trust in the media organization and the perceived reliability of the information it provides (Moran and Nechushtai, 2022). Research on trust building indicates that in digital environments, the identity cues of an authoritative platform has serve as a crucial signal for establishing credibility (Tandoc et al., 2018). To examine how source credibility shapes belief updating, this study focused on the dimension of institutional authority. This concept captures the influence a publication derives from its official status, its recognized role within a media institution, and the broad public trust it consequently holds (Tandoc et al., 2018). To represent different levels of such authority, two contrasting news sources were selected: People’s Daily and Jiupai News. This choice is grounded in empirical media credibility research in China, which documents a pronounced “stronger central, weaker local” credibility gradient, where central-level outlets consistently hold a significant trust advantage over local ones (Yang et al., 2026; Qiang and Hu, 2025). People’s Daily exemplifies a central-level, high-authority source. In contrast, Jiupai News is a market-oriented online platform operated by a local press group, aligning with the “weaker local” credibility category. The core contrast between these sources lies in their fundamental institutional position and perceived authority, not in specific content. This study design enables specific isolation to test the effect of institutional authority on how people process fact-checking information.

Traditional research on rumor denial has heavily relied on self-reported measures and behavioral outcomes, such as whether people believe or share a corrected claim (Pal et al., 2019; Li and Sakamoto, 2014; Moran and Nechushtai, 2022). These studies observe what decisions are made, but offer limited insight into the underlying cognitive processes. When analysis is limited to final choices, real-time cognitive dynamics, such as conflict monitoring or trust calibration, remain largely unexplored. Elucidating these processes requires methods that can capture the brain’s activity in real time while judgments are formed. Psychophysiological tools, such as brain imaging tools, have received growing attention due to their ability to complement other sources of data, including self-reported data (i.e., questionnaires to investigate rumors behavior and rumors belief) (Pennycook et al., 2020; Ding et al., 2024). Prior neuroscience work on credibility has largely focused on different questions, using electroencephalography (EEG) or functional magnetic resonance imaging (fMRI) to study memory-based truth judgment or the continued influence of corrected misinformation (Düzel et al., 1997; Gordon et al., 2019). Direct analysis of the neural correlates of rumor cognition remains scarce (Ding et al., 2024; Gordon et al., 2019; Kawiak et al., 2020; McClellan et al., 2026).

In contrast to other methods, functional near-infrared spectroscopy (fNIRS) offers unique non-invasive monitoring advantages for unraveling the mysteries of cognitive processing in the brain (Quaresima and Ferrari, 2019). fNIRS precisely captures dynamic changes in oxy-hemoglobin (HbO) and deoxy-hemoglobin (HbR) concentrations within the cerebral cortex, providing real-time feedback through these biomarkers (Herold et al., 2018). Due to its non-invasiveness, high temporal resolution, and good spatial localization capabilities, fNIRS has emerged as an essential tool in cognitive science (Waight et al., 2024; Chandra and Choudhury, 2023). fNIRS is well-suited for this purpose, as it enables the measurement of neural engagement in realistic tasks, focusing on the prefrontal cortex (PFC), a brain area vital for critical thinking, evaluating credibility, and resolving conflicting information (McClellan et al., 2026; Quaresima and Ferrari, 2019). Similar to functional magnetic resonance imaging (fMRI), fNIRS accurately detects and quantifies subtle variations in regional hemoglobin concentrations within the brain, allowing the analysis of specific areas following activation by cognitive tasks. These changes directly reflect neural activity intensity and offer intuitive and robust evidence for understanding dynamic interactions within brain functional networks, as well as the neural underpinnings of cognitive processes (Zinos et al., 2024). Moravec et al. utilized EEG and found that a fake news label on a headline did not influence veracity judgment, and that labeling headlines as false did not influence users’ beliefs (Moravec et al., 2019). Another study by McClellan et al. (2026) reported increased activation in the PFC when participants were exposed to misinformation that conflicted with their attitudes. Despite the broad adoption of fNIRS (Quaresima and Ferrari, 2019), its application in exploring how source authority shapes the brain’s response during fact-checking is novel.

Following Moravec et al. (2019), our study is grounded in cognitive dissonance theory and examines how it influences behavior in online rumors. As stated by Moravec et al., this situation can create cognitive dissonance, where a user is inclined to believe a fake headline, despite it being labeled as false. According to cognitive dissonance theory, individuals experience psychological discomfort when confronted with information that contradicts their existing beliefs or initial judgments (Festinger, 1962). To resolve this discomfort, an intuitive, fast response or analytical processing may be triggered to reduce this dissonance through belief adjustment or source discounting (Moravec et al., 2019; Kahneman, 2012). When the issue is perceived as low in personal relevance, dissonance is often minimized or dismissed, defaulting to their prior beliefs (Nickerson, 1998). However, when motivated, individuals invest additional cognitive resources to reconcile the contradiction—a process that demands more time and mental effort (Festinger, 1962). In summary, this study posits that false or true labels assigned to rumors that are aligned or misaligned with a person’s beliefs by authoritative vs. non-authoritative parties will trigger cognitive dissonance.

To elucidate these neurocognitive mechanisms, fact-checking sources were operationalized along the dimension of authority (authoritative vs. non-authoritative), and their interactions with information veracity (true vs. false) were examined. Within the framework of cognitive dissonance, this study hypothesizes that corrections from a high-authority source may trigger a stronger initial neural conflict signal, but also a quicker pathway to belief updating to resolve discomfort. Conversely, corrections from a low-authority source might induce a different pattern of neural engagement, potentially eliciting more sustained evaluative processing. Using fNIRS, participants’ judgment accuracy and changes in PFC activation were monitored following different fact-checking prompts. By exploring the relationship between fact-checker authority and real-time cognitive processing in the brain, this study aims to reveal the neural underpinnings of how authority shapes dissonance resolution and decision-making. The findings provide neuroscientific evidence to inform strategies for effective communication and trust calibration in digital information environments.

## Materials and methods

The study was approved by the Ethics Committee of the Institute of Neuroscience and Cognitive Psychology of Anhui Polytechnic University (AHPU-SEM-2021-002) and conducted under the ethical standards of the Declaration of Helsinki. All participants voluntarily signed the informed consent form after reading and fully comprehending its contents.

### Participants

An *a priori* power analysis was conducted using G*Power (Faul et al., 2007) to determine the requisite sample size. Based on the study design, repeated ANOVA with two within-subject factors (source and label, each with two levels) was used to calculate the sample size. A target sample of 36 participants was determined based on an effect size of 0.25, an alpha level (α) of 0.05, and a statistical power (1 − β) of 0.95. To ensure data integrity and experimental reliability, 42 participants (19 females, 23 males; mean age = 22.4 years, SD = 2.59 years) were recruited. The subjects were undergraduate or graduate students with relevant academic backgrounds and experimental competence. First, the participants’ health status was screened to confirm that none suffered from neurological or psychiatric disorders, ensuring normal vision or appropriate correction. Before starting the experiment, the experimental procedures and the fundamental principles of fNIRS technology were thoroughly explained to all participants.

### Stimulus material and experimental design

Initially, 24 pieces of information were selected from current social media, covering two common themes: health and technology. A total of 111 undergraduate students (58 male, 53 female, Mage = 22.0 years, SD = 2.3) were recruited to participate in a pre-experiment. All participants evaluated the authenticity (true/false) of these pieces of information. The results showed that the overall judgment accuracy (45.31% for true information, 54.17% for false information) approximated chance performance, indicating that the information possessed substantial ambiguity in terms of truthfulness and was suitable for investigating the influence of external sources. Based on these results, four representative pieces of true information and four pieces of false information were selected, ultimately forming a set of 8 core experimental materials. To control for potential confounding variables, quantitative balance checks on the final 8 selected materials were conducted. An independent-samples *t*-test revealed no significant difference in the average word count between the true and false information (*M*_true_ = 18.75 words, SD = 1.26; *M*_false_ = 17.75 words, SD = 0.96; *t*(6) = 1.265, *p* = 0.253).

This study employed E-Prime 3.0 software to develop the experimental protocol. The materials used and experimental protocol can be downloaded from https://pan.baidu.com/s/1xl-RywO_TFppjtelTM5LWg?pwd=acdi. Previous research reported that the cognitive processing differs fundamentally between statements containing true versus false conceptual features (Marques et al., 2009). Therefore, in the experimental design, and to ensure the objectivity and accuracy of the results, two pieces of true information were randomly assigned the label of authoritative third-party fact-checkers (People’s Daily), whereas the other two pieces of true information were assigned the label of non-authoritative third-party fact-checkers (Jiupai News). The same manipulation was applied to the false information to maintain balance across the experimental conditions. The authority of a news source relies primarily on the public’s high degree of trust in the media organization (Moran and Nechushtai, 2022). Hence, the identity marker of an authoritative platform serves as a key trust signal (Tandoc et al., 2018). Recent studies have shown a significant difference in authority between local and central media (Yang et al., 2026; Qiang and Hu, 2025). These findings also confirm the successful operationalization of source authority in our experiment.

Before the formal experiment, all participants completed a pre-experiment practice session. This session consisted of 4 distinct stimulus materials, presented sequentially and repeated only once. The practice session included one material for each of the two information types and one for each information source, using independent materials not included in the formal experiment. During the practice, each stimulus was presented for 5 s, which then disappeared automatically. Within these 5 s, participants were required to judge the authenticity of the labeled information. After the participant’s response, the experimenter provided oral feedback to ensure they understood the task requirements. The goal of this experimental paradigm was to capture the hemodynamic response associated with processing information from different sources. The experiment employed a block design, presenting a total of 16 information items. Block 1 contained 4 true information items and 4 false information items. In block 2, among the 4 true information items, two were labeled as from People’s Daily and judged as true, and two were labeled as from Jiupai News and judged as true; among the 4 false information items, two were labeled as from People’s Daily and judged as false, and two were labeled as from Jiupai News and judged as false. Throughout the experiment, Block 1 and Block 2 were presented in sequence, with the items within each block randomized.

**Figure 1:**
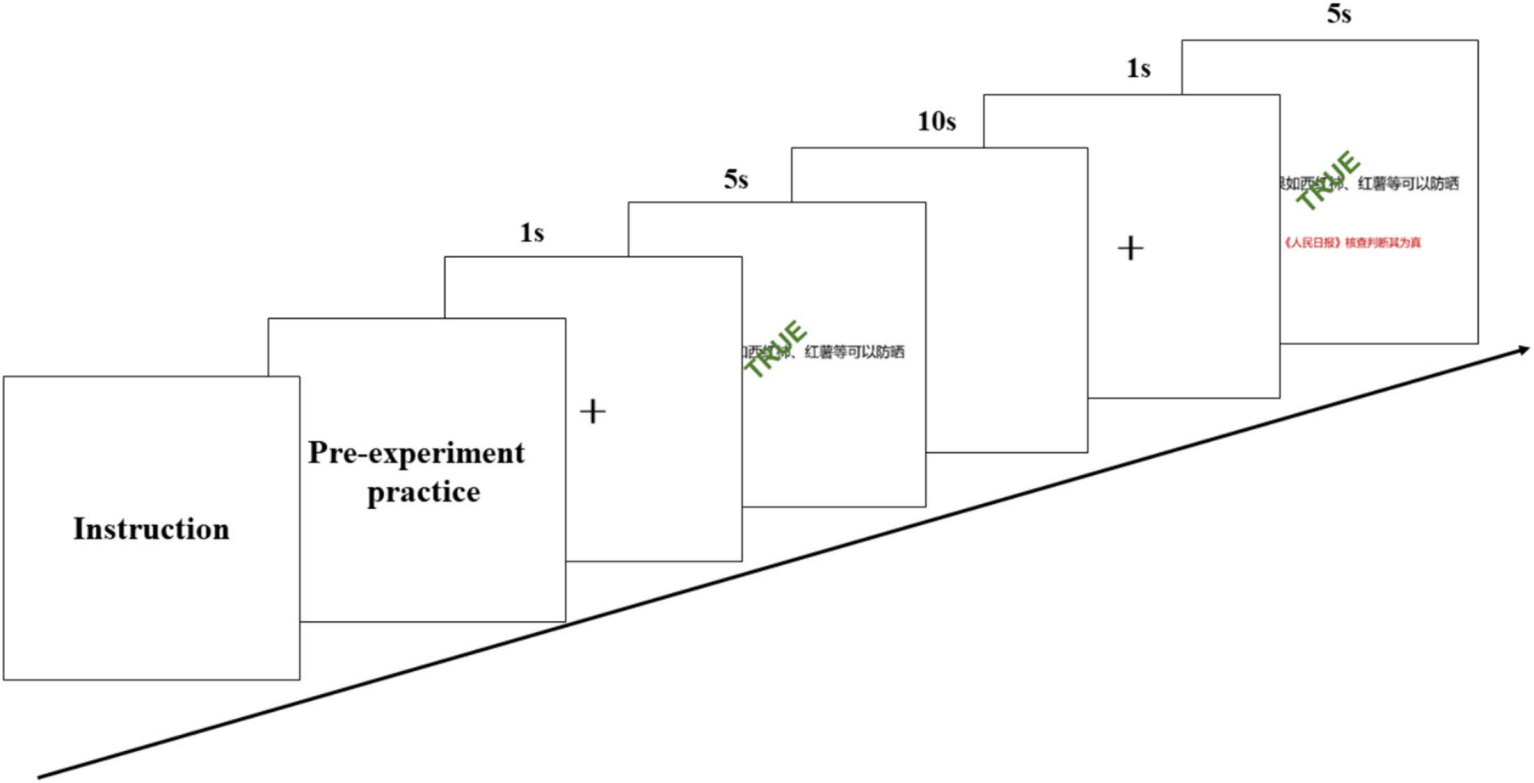
The experimental procedure.

As shown in Figure 1, each stimulus followed a structured timeline. The information was presented for 5 s, during which participants were required to read and judge its authenticity within the five-second window. The participants responded by pressing the number 0 on the keyboard for information deemed true, and the number 1 for information deemed false. Considering that the standard hemodynamic response requires approximately 10 s after reaching its peak to return to baseline levels (Kaiser et al., 2014; Leff et al., 2006; Malonek and Grinvald, 1996), a 10-s blank rest interval was allowed after each judgment. Before the next trial, a fixation cross was displayed on the screen for 1 s. To obtain a robust hemodynamic response for signal averaging, each unique combination was presented five times in random order within its respective condition block. Throughout the experiment, fNIRS data were continuously recorded for approximately 30 min per participant, ensuring data completeness and accuracy, and laying a solid foundation for subsequent data analysis and research. The raw data can be downloaded from https://cstr.cn/31253.11.sciencedb.35305.

### Data acquisition

The study employed the NIRSport 2 near-infrared spectroscopy (NIRS) brain functional imaging system (Nanjing Jian Chuang Technology Co., Ltd., China), which synergized LED light sources and advanced active detector technology for wearable brain functional imaging. The NIRSport 2 not only achieves exceptional portability but also significantly enhances the flexibility and precision of experimental design through its built-in standardized probe positioning mechanism and flexible custom configuration capabilities. The system’s multiple digital I/O trigger options ensure precise capture and recording of triggering events, laying a solid foundation for the timeliness and accuracy of experimental data. Furthermore, its real-time data stream display function allows researchers to monitor the experimental progress instantly and adjust experimental parameters, ensuring research efficiency. Notably, the NIRSport 2 demonstrates remarkable compatibility, seamlessly integrating with diverse brain functional imaging approaches such as EEG, fMRI, TMS (transcranial magnetic stimulation), and eye-tracking systems (Li et al., 2022; Esposito et al., 2020; Zhu and Lv, 2023). This enables simultaneous acquisition and analysis of multimodal data, offering unprecedented insights into the complex functional networks of the brain.

**Figure 2:**
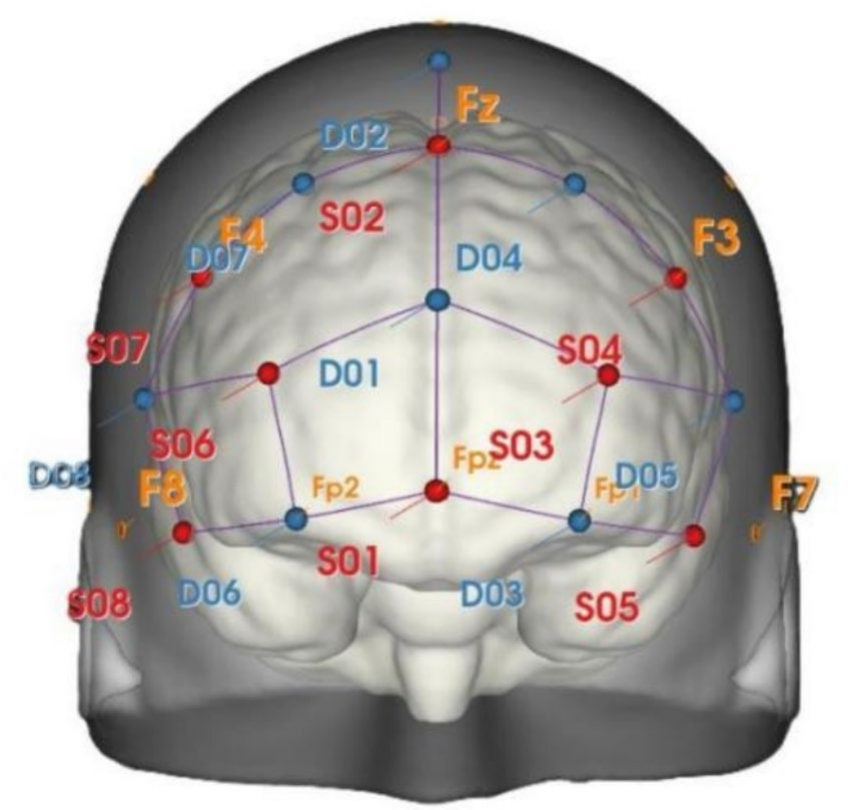
Distribution of light sources and detectors distribution.

This study utilized the NIRSport 2 system to collect hemodynamic signals from the PFC. The system configuration consisted of 8 light sources and 8 detectors, forming a total of 23 effective measurement channels. The distance between the light sources and detectors was strictly controlled at 30 mm to achieve an optimal balance between signal penetration depth and spatial resolution. The sampling frequency was set at 10.17 Hz. The probe array was positioned according to the international 10–20 EEG placement system. Specifically, a detector in the central column was fixed at the Fpz point, serving as the reference landmark. The entire probe array symmetrically covered the bilateral prefrontal regions, ensuring complete coverage of the target Brodmann areas (BA 8, 9, 10, 11, 45, 46). Collectively, these regions constitute the core of the prefrontal cortex, playing pivotal roles in executing high-level cognitive functions, including but not limited to thought and intuition processing, encoding and retrieval of memory information, and formulation of problem-solving strategies. They are intimately connected with the limbic system, jointly modulating individuals’ behavior (Panagiotaropoulos, 2024). The probe layout is detailed in Figure 2, and the corresponding Brodmann areas for each channel are listed in Table 1. Prior to the formal experiment, each participant wore the head cap, and calibration was performed using the system’s built-in signal quality check function. This process displayed the signal intensity of each channel in real-time. The contact pressure between the probes and the scalp was adjusted until the raw light intensity signals of all channels stabilized and the signal-to-noise ratio reached an acceptable level, after which formal data recording commenced.

**Table 1:** MNI coordinates of fNIRS channels.

Channel	Brodmann area (BA)	MNI coordinates			Overlap	ROI assignment
		X	Y	Z		
Ch1	10 - Frontopolar area	2.667	68.667	13.333	1	FP-ROI
Ch2	11 - Orbitofrontal area	−11.667	73	−4.333	0.556	OFC-ROI
Ch3	11 - Orbitofrontal area	14.667	73	−4.667	0.583	OFC-ROI
Ch4	9 - Dorsolateral prefrontal cortex	1.667	55.333	40.333	0.909	DLPFC-ROI
Ch5	8 - Includes frontal eye fields	1.667	30.667	59	1	NONE
Ch6	9 - Dorsolateral prefrontal cortex	−10.667	46.333	51.667	0.775	DLPFC-ROI
Ch7	9 - Dorsolateral prefrontal cortex	13.333	46.667	52.333	0.776	DLPFC-ROI
Ch8	10 - Frontopolar area	−14	68	23.667	1	FP-ROI
Ch9	10 - Frontopolar area	−25.333	68.667	3.667	0.625	FP-ROI
Ch10	46 - Dorsolateral prefrontal cortex	−24.667	56.667	34.333	0.506	DLPFC-ROI
Ch11	46 - Dorsolateral prefrontal cortex	−42	55.667	15	0.832	NONE
Ch12	9 - Dorsolateral prefrontal cortex	−29.667	44.667	42.667	0.876	DLPFC-ROI
Ch13	45 - Pars triangularis Broca’s area	−47	44.667	23.667	0.759	NONE
Ch14	11 - Orbitofrontal area	−34	64.333	−9.333	0.469	OFC-ROI
Ch15	46 - Dorsolateral prefrontal cortex	−48.667	50	−1	0.911	NONE
Ch16	10 - Frontopolar area	17.333	68.333	24.667	1	FP-ROI
Ch17	10 - Frontopolar area	29.333	69	4.333	0.656	FP-ROI
Ch18	9 - Dorsolateral prefrontal cortex	27.333	56.667	34.667	0.461	DLPFC-ROI
Ch19	46 - Dorsolateral prefrontal cortex	45.333	54.667	16.333	0.852	DLPFC-ROI
Ch20	9 - Dorsolateral prefrontal cortex	33.667	44.667	42.667	0.857	DLPFC-ROI
Ch21	45 - Pars triangularis Broca’s area	50	42.667	25.667	0.894	NONE
Ch22	11 - Orbitofrontal area	37.667	64.667	−9.333	0.403	OFC-ROI
Ch23	46 - Dorsolateral prefrontal cortex	51.333	49.667	0.667	0.849	NONE

The acquired raw light intensity data were preprocessed using the Homer2 toolbox (MATLAB) with the following steps:

1. Raw Data Inspection: The Homer2 toolkit was used to perform an initial check on the acquired raw NIRS data to ensure data integrity and quality, excluding obvious anomalies or corrupted data.
2. Conversion of Light Intensity to Optical Density (OD): The raw light intensity data were converted to optical density values.
3. Motion Artifact Detection and Correction: The Spline interpolation method was employed for motion artifact detection and correction on the OD data. By identifying abnormal jump points in the signal, Spline interpolation was used to smooth the artifact-affected regions, effectively reducing the impact of motion artifacts on data quality.
4. Bandpass Filtering: Bandpass filtering was applied to the corrected OD data. The filtering range was typically set from 0.01 Hz to 0.5 Hz to remove low-frequency drifts (e.g., instrumental drift or physiological low-frequency noise) and high-frequency noise (e.g., cardiac or respiratory interference), preserving the effective signal components related to brain activity.
5. Conversion of Optical Density Back to Light Intensity: The filtered OD data were converted back to light intensity data for subsequent analysis and visualization. This step provides standardized data for subsequent signal averaging and statistical analysis.

### Definition and rationale for region of interest selection

Based on the core functional localization of the prefrontal cortex (PFC) in “information credibility assessment,” “cognitive conflict monitoring,” and “decision-making control” (Panagiotaropoulos, 2024), and combined with the core research question of this study, the regions of interest (ROIs) in this study were defined through the following two steps: First, with reference to the corresponding relationship between the fNIRS probe layout and Brodmann areas (Table 1), functional subregions within the PFC that are directly related to “credibility judgment” and “conflict resolution” were identified. Second, drawing on reports of brain regions associated with “information source evaluation” in previous neuroimaging studies (Kawiak et al., 2020; McClellan et al., 2026), channels irrelevant to the cognitive processes of this study were excluded, ultimately forming 3 core ROIs. The details are as follows:

#### Dorsolateral prefrontal cortex ROI

The dorsolateral prefrontal cortex (DLPFC, corresponding to Brodmann areas 9/46, BA9/46) is a core brain region for cognitive control and working memory integration, responsible for logical analysis, conflict resolution, and adjustment of decision-making strategies in the judgment of information authenticity. In this study, this region is involved in the “matching assessment between the authority cues of fact-checkers and the authenticity of information,” and serves as a key region for verifying the hypothesis that “authoritative sources improve judgment accuracy by reducing cognitive conflict.” According to the corresponding relationship between channels and Brodmann areas in Table 1, the dorsolateral prefrontal cortex ROI (DLPFC-ROI) includes the following 8 channels: Ch4 (BA9), Ch6 (BA9), Ch7 (BA9), Ch10 (BA46), Ch12 (BA9), Ch18 (BA9), Ch19 (BA46), and Ch20 (BA9).

#### Frontopolar area ROI

The frontopolar area (corresponding to Brodmann area 10, BA10) is responsible for handling the “conflict coordination between information source credibility and content authenticity.” Especially when “external cues (e.g., source authority) are inconsistent with internal cognition (e.g., prior beliefs),” this region regulates the information integration process by enhancing activation (Panagiotaropoulos, 2024). In this study, this region is involved in the “processing of cognitive uncertainty induced by non-authoritative sources” and is a key region for explaining the phenomenon that “non-authoritative sources achieve higher judgment accuracy for false information.” According to Table 1, the frontopolar area ROI (FP-ROI) includes the following 5 channels: Ch1 (BA10), Ch8 (BA10), Ch9 (BA10), Ch16 (BA10), and Ch17 (BA10). Moravec et al. (2019) found in a study on false information processing that the activation of the frontopolar area is significantly correlated with the “vigilance assessment of low-credibility sources.”

#### Orbitofrontal cortex ROI

The orbitofrontal cortex (corresponding to Brodmann area 11, BA11) is involved in emotional credibility assessment and decision-making bias regulation, and is responsible for transforming the “subjective trust in source authority” into specific judgment behaviors (Feng et al., 2023). In this study, this region is involved in the “trust transfer process toward People’s Daily (a high-authority source)” and is a key region for explaining the phenomenon that “authoritative sources improve the judgment accuracy of true information.” According to Table 1, the Orbitofrontal Cortex ROI (OFC-ROI) includes the following 4 channels: Ch2 (BA11), Ch3 (BA11), Ch14 (BA11), and Ch22 (BA11). Moore et al. (2021) confirmed through fMRI studies that the activation intensity of the orbitofrontal cortex is positively correlated with the “degree of trust in official media.”

All statistical analyses of fNIRS data in this study (including Friedman test and Nemenyi post-hoc test) were limited to the 17 channels of the above three ROIs. Among the total 23 channels, 6 channels (Ch5/Ch11/Ch13/Ch15/Ch21/Ch23) were excluded as they are irrelevant to the cognitive processes of this study. None of these channels are associated with the “impact of source authority on rumor processing” (the core focus of this study). This ROI definition strategy ensures the “functional specificity” and “theoretical relevance” of statistical analyses, avoids the risk of false positives caused by undifferentiated analysis of all channels, and is highly consistent with the hypothetical framework of this study based on cognitive dissonance theory.

## Results

### Participant performance and subjective rating

This study conducted a thorough statistical analysis of the participants’ behavioral data, aiming to evaluate their accuracy in judging information. To ensure the rigor and precision of our analysis, the chi-square test was employed as a statistical method. The chi-square test is particularly suited for determining the presence of significant differences in the distribution of data across different groups regarding a specific characteristic (Franke et al., 2012). Participants’ judgment accuracy data were analyzed using chi-square tests of independence. This non-parametric test was selected due to the data consisting of frequency counts of categorical outcomes (i.e., the number of “true” vs. “false” judgments) under different experimental conditions (e.g., different source labels). The chi-square test is the appropriate method for determining whether the distribution of these categorical responses is independent of the experimental conditions. This approach directly addresses our primary behavioral research question: whether the source of fact-checking influences the proportion of “true” judgments. Before the analysis, the assumptions underlying the chi-square test were verified, including that all expected cell frequencies were greater than five.

#### Accuracy in identifying true information

In the absence of third-party fact-checker (T-NS) labeling, participants’ accuracy in assessing true information was merely 37%. However, upon introducing non-authoritative third-party fact-checkers (T-JS), specifically Jiupai News as the fact-checker, the participants’ accuracy improved to 45%. Notably, upon labeling with authoritative third-party fact-checkers (T-RS), namely People’s Daily, the participants’ accuracy in judging true information significantly increased to 62%. A chi-square test analysis revealed a significant main effect of the source condition on judgment accuracy for true information, χ^2^(2, *N* = 264) = 11.092, *p* = 0.004.

These findings indicated that the inclusion of third-party fact-checkers enhances participants’ accuracy in information judgment, with the addition of authoritative third-party fact-checkers significantly increasing the accuracy and credibility of information. Furthermore, the accuracy of authoritative third-party fact-checkers (People’s Daily) was significantly higher than that of non-authoritative third-party fact-checkers (Jiupai News). This difference confirmed the profound impact of the authority of third-party fact-checkers on participants’ judgments (Xu et al., 2023).

#### Accuracy in identifying false information

In the absence of third-party fact-checkers (F-NS), participants’ accuracy in judging false information was 36%. However, upon introducing third-party fact-checkers, a significant improvement in judgment accuracy was observed. Specifically, labeling with a non-authoritative third-party fact-checkers (F-JS, Jiupai News) resulted in an increase to 59% in the accuracy of recognizing false information. Similarly, labeling with authoritative third-party fact-checkers (F-RS, People’s Daily) resulted in an increase to 55% in the accuracy of recognizing false information. However, this increase was slightly lower than the non-authoritative counterpart.

The results of this study highlight a crucial finding. In the context of false information, both the introduction of authoritative and non-authoritative third-party fact-checkers effectively and significantly enhanced participants’ judgment accuracy. This discovery contradicts the pattern observed in the processing of true information, where the addition of either authoritative or non-authoritative third-party fact-checkers exerted no notable difference in judgment accuracy. This phenomenon may be explained by the cognitive dissonance theory (Metzger et al., 2020), suggesting that varying levels of authority in third-party fact-checkers elicit distinct psychological adjustments while identifying and processing false information.

### Hemodynamic responses

All statistical analyses of hemodynamic responses were restricted to three predefined regions of interest (ROIs), including the dorsolateral prefrontal cortex (DLPFC)-ROI (Channels Ch4/Ch6/Ch7/Ch10/Ch12/Ch18/Ch19/Ch20), the frontopolar (FP)-ROI (Channels Ch1/Ch8/Ch9/Ch16/Ch17), and the orbitofrontal cortex (OFC)-ROI (Channels Ch2/Ch3/Ch14/Ch22). The selection of these ROIs was based on the theory of prefrontal cortex functional partitioning and previous studies on information source credibility assessment. This ensured that the analysis focused on brain regions directly associated with “source authority judgment” and “cognitive conflict resolution,” while excluding irrelevant channels to avoid interference with the results. This study relies on the quantification of changes in HbO, which serves as a critical indicator for estimating dynamic alterations in cerebral blood flow (CBF) within activated brain regions (Meek, 2002). In the data acquisition process, an optimized Beer–Lambert law approach was employed to accurately extract the concentration changes of oxyhemoglobin from the raw signals (Chance et al., 1998). To enhance data reliability, the Homer2 software package was utilized (Huppert et al., 2009), converting light signal intensities into optical density (OD) values. Subsequently, detected motion artifacts were meticulously removed using Spline interpolation, ensuring that the data remained unaffected by non-physiological factors. To further refine signal quality, bandpass filtering was implemented, which effectively suppressed noise components while preserving signal frequencies pertinent to physiological variations. Upon completion of these preprocessing steps, the OD values were converted into concentration values, and block averaging was performed, aiming to mitigate random errors and enhance the statistical robustness of the data. Finally, comprehensive data analyses were conducted within the MATLAB software environment, comprehensively elucidating the relationship between HbO variations and brain functional activities. As fNIRS measures infrared light intensity, multiple signal-processing and conversion steps were required to transform the raw intensity data into final measurements, reported in micromolar (μM) concentrations.

#### Effect of third-party fact-checkers on the evaluation of authentic information

This study employed the Friedman test to investigate whether statistically significant differences exist among multiple paired quantitative data across three channels: T-NS, T-JS, and T-RS. This non-parametric test was selected for the following reasons: Firstly, the data structure represented a repeated-measures design (the same participant underwent all conditions), with a continuous dependent variable (the change in HbO concentration). To determine whether the data were suitable for parametric tests (repeated-measures ANOVA), the Shapiro–Wilk test was first conducted on the difference scores to assess normality. The test results indicated that the data from a considerable number of channels violated the assumption of normal distribution. Consequently, the Friedman test was adopted as a robust non-parametric alternative to examine whether differences exist among multiple related samples (i.e., different source conditions). This method does not rely on specific assumptions regarding the shape of the data distribution.

To control the family-wise error rate associated with multiple pairwise comparisons within each channel, post-hoc pairwise comparisons between the three conditions (T-NS, T-JS, T-RS) were conducted using the Nemenyi test when the Friedman test yielded a significant result for a given channel. The *p*-values from these Nemenyi comparisons were then adjusted using the Bonferroni correction method to account for the three comparisons performed per channel. This approach ensured that the overall Type I error rate for the set of post-hoc tests within each channel was maintained at α < 0.05.

The experimental data were meticulously analyzed, as presented in Table 2. Statistically significant differences were observed among T-NS, T-JS, and T-RS on specific channels, including Ch5 (*p* = 0.007 < 0.01), Ch6 (*p* = 0.049 < 0.05), Ch7 (*p* = 0.030 < 0.05), Ch18 (*p* = 0.027 < 0.05), Ch20 (*p* = 0.042 < 0.05), and Ch21 (*p* = 0.017 < 0.05). The specific magnitude of these differences was further analyzed by comparing the medians of the data across each channel.

**Table 2:** Results of multi-sample Friedman analysis for true information.

Name	Median	Statistical χ^2^ value	*p*	Median difference	Median difference	*p*
NS Ch5	0.058	9.905	0.007**	NS-JS	0.039	0.006**
JS Ch5	0.019			NS-RS	0.002	0.644
RS Ch5	0.057			JS-RS	−0.038	0.074
NS Ch6	0.071	6.048	0.049*	NS-JS	0.041	0.074
JS Ch6	0.029			NS-RS	−0.004	0.900
RS Ch6	0.075			JS-RS	−0.046	0.095
NS Ch7	0.039	7.000	0.030*	NS-JS	0.017	0.057
JS Ch7	0.022			NS-RS	−0.015	0.900
RS Ch7	0.054			JS-RS	−0.032	0.057
NS Ch18	0.032	7.190	0.027*	NS-JS	0.061	0.032*
JS Ch18	−0.029			NS-RS	0.014	0.894
RS Ch18	0.017			JS-RS	−0.047	0.095
NS Ch20	0.041	6.333	0.042*	NS-JS	−0.001	0.043*
JS Ch20	0.042			NS-RS	−0.027	0.831
RS Ch20	0.067			JS-RS	−0.025	0.152
NS Ch21	0.039	8.190	0.017*	NS-JS	0.027	0.013*
JS Ch21	0.013			NS-RS	0.014	0.188
RS Ch21	0.025			JS-RS	−0.012	0.519

Ch5 and Ch21 are non-ROI channels. Their results are for reference only and not included in the core cognitive interpretation. *p < 0.05, **p < 0.01.

On Channel Ch5 (ROI Assignment: None, BA8, involved in eye movement control), the difference between T-JS and T-RS was non-significant (*p* = 0.074 > 0.05). As this channel is irrelevant to the core cognitive processes of source authority assessment, this non-significant result further confirms no reliable distinction in HbO concentration between non-authoritative and authoritative fact-checking conditions for true information. On Channel Ch6 (DLPFC-ROI, BA9), the difference between T-NS and T-JS was non-significant (*p* = 0.074 > 0.05), which suggests non-authoritative fact-checking does not meaningfully modulate neural activity related to cognitive control (the core function of DLPFC-ROI) relative to the no-source condition. On Channel Ch7 (DLPFC-ROI, BA9), the differences between T-NS and T-JS (*p* = 0.057 > 0.05) and between T-JS and T-RS (*p* = 0.057 > 0.05) were both non-significant. Even though the uncorrected *p*-values were close to the 0.05 threshold, the lack of statistical significance confirms that variations in HbO concentration between conditions reflect random neural variability rather than systematic differences in cognitive processing induced by fact-checker authority. However, no significant difference between T-NS and T-RS was found. On both Channels Ch18 and Ch20, significant differences were found between T-NS and T-JS (*p* = 0.032 < 0.05 and *p* = 0.043 < 0.05, respectively), while no significant differences were detected with other comparison groups. On Channel Ch21, a significant difference was evident between T-NS and T-JS (*p* = 0.013 < 0.05), with no significant differences observed between other comparison groups.

Specifically, within the context of true information, the introduction of non-authoritative third-party fact-checkers was associated with a compelling pattern of neural activity modulation. In the absence of third-party fact-checkers (NS condition), a significant task-related HbO increase was observed, suggesting a pronounced activation state. However, labeling with non-authoritative third-party fact-checkers (JS condition) led to a marked decrease in HbO across multiple critical channels within the rostro-prefrontal cortex (RPFC). This statistically significant difference underscores the substantial disruptive effect that non-authoritative third-party fact-checkers exert on the brain’s processing mechanisms as participants engage with authentic information. This contributes to our understanding of the interplay between information verification and cognitive load.

#### Effects of third-party fact-checkers on the evaluation of false information

In this study, statistical analyses were conducted on three sets of data, namely F-NS, F-JS, and F-RS, across multiple channels (Ch19, Ch13, Ch17). Table 3 presents a detailed discussion of the analytical findings.

**Table 3:** Results of a multi-sample Friedman analysis for false information.

Name	Median	Statistical χ^2^ value	*p*	Median difference	Median difference	*p*
NSCh19	0.039	8.333	0.016*	200–201	0.021	0.074
JSCh19	0.018			200–202	0.043	0.018*
RSCh19	−0.004			201–202	0.022	0.831
NSCh13	0.043	5.190	0.075	200–201	−0.005	0.900
JSCh13	0.049			200–202	0.041	0.095
RSCh13	0.003			201–202	0.046	0.152
NSCh17	0.027	5.190	0.075	200–201	0.025	0.152
JSCh17	0.003			200–202	0.027	0.095
RSCh17	0.001			201–202	0.002	0.900

Ch13 is non-ROI channels. The result is for reference only and not included in the core cognitive interpretation. *p < 0.05.

Comparisons among F-NS, F-JS, and F-RS on Channel Ch19 revealed significant differences. Specifically, the comparison between F-NS and F-RS on Ch19 exhibited a significant difference (*p* = 0.016 < 0.05). Additionally, on Channel Ch19 (DLPFC-ROI, BA46, core region for cognitive conflict resolution), the comparison between F-NS and F-JS was non-significant (*p* = 0.074 > 0.05). This result indicates no reliable difference in HbO concentration between the no-source and non-authoritative fact-checking conditions for false information, suggesting non-authoritative sources do not modulate cognitive conflict resolution processes in the DLPFC-ROI. In contrast, the comparison between F-JS and F-RS on Ch19 demonstrated no significance. Similar non-significant results were observed on Channels Ch13 and Ch17. On Channel Ch13 (ROI Assignment: None, BA45, involved in language processing), comparisons among F-NS, F-JS, and F-RS were non-significant (*p* = 0.075 > 0.05); as this channel is unrelated to source authority assessment, no further interpretation was conducted. On Channel Ch17 (FP-ROI, BA10), comparisons among F-NS, F-JS, and F-RS were also non-significant (*p* = 0.075 > 0.05). The *p*-value < 0.1 does not meet the conventional statistical significance threshold (α = 0.05) for cognitive neuroscience research, so this result does not support follow-up claims about false information processing.

For pieces of false information only (NS condition), a significant task-related HbO increase was seen, indicative of a pronounced activation state. However, when authoritative third-party fact-checkers were integrated into the false information (RS condition), a marked decrease in HbO was observed within the RPFC on specific channels, such as Ch19. This finding further substantiates the influence of fact-checking authority on neural processing during the evaluation of false information.

#### Hemodynamic activation

**Figure 3:**
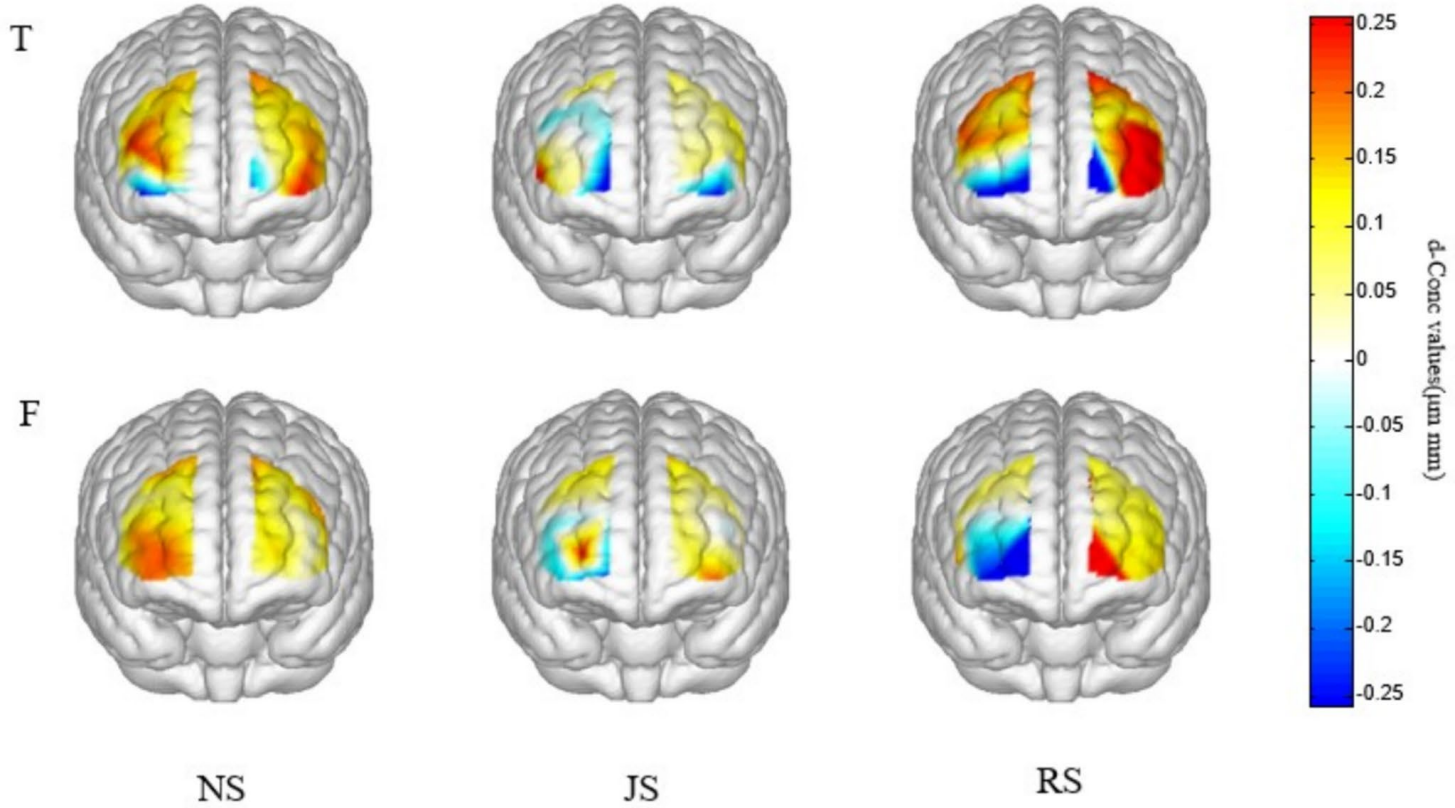
Visualization of AOF activation in three cases.

To investigate the specific impacts of third-party fact-checking on individual cognitive processes, this study further compared cortical activation patterns before and after the incorporation of fact-checking across different types of information. The present study unveiled the pronounced influence of third-party fact-checkers on cerebral hemodynamics, particularly the concentration of HbO. Figure 3 illustrates the variations in HbO concentration following the addition of distinct third-party fact-checkers to different information types.

Specifically, within the context of true information, the introduction of non-authoritative third-party fact-checkers elicited a compelling neural activity pattern. In the absence of any additional fact-checking (NS condition), the RPFC, which is a crucial brain region responsible for cognitive control, decision-making, and information evaluation (Miller and Cohen, 2001), exhibited a significant activation state. This activation is inferred to reflect the brain’s active engagement in cognitive conflict monitoring and deliberative assessment of information veracity, such as detecting inconsistencies between the information and prior knowledge frameworks (McClellan et al., 2026). However, upon transitioning to the scenario with non-authoritative third-party fact-checkers (JS condition), a statistically significant decrease in RPFC activation intensity was observed across multiple key channels (including Ch5, Ch6, Ch7, Ch18, Ch20, and Ch21). This finding suggests that non-authoritative fact-checking information may interfere with the brain’s default evaluation of authentic information. Combined with the behavioral data showing only a marginal and non-significant increase in accuracy, this reduction in neural activity likely does not reflect an enhancement of a “deliberative assessment mechanism” (Moravec et al., 2019). Instead, the results more plausibly indicate that participants reduced their cognitive investment in the information or adopted a heuristic processing strategy based on the perceived low credibility of the source, engaging in rapid “cognitive offloading.” Consequently, this shift in processing strategy did not yield significant gains in behavioral accuracy.

Similarly, the impact of third-party fact-checkers on RPFC activation patterns was determined for pieces of false information. In the presence of false information only (NS condition), RPFC activation persisted, albeit potentially reflecting the brain’s initial skepticism or assessment process toward information veracity, as compared to activation by authentic information. Specifically, the detection of potential discrepancies between the false information and existing knowledge structures, which triggers cognitive conflict signals that prompt further evaluation (McClellan et al., 2026). Notably, when authoritative third-party fact-checkers were integrated into false information (RS condition), a marked reduction in RPFC activation was observed on specific channels, such as Ch19, strongly suggesting that authoritative fact-checking may serve as a “fast track” in misleading contexts (Kahneman, 2012), This “fast track” effect manifests as participants relying on the high credibility of the source to resolve initial cognitive conflict (Moore et al., 2021), thereby engaging in less intensive cognitive deliberation and assessment of the information itself, which accelerates decision-making but may reduce the depth of verification regarding information actual veracity.

In-depth analysis of the experimental results revealed that for authentic information, the integration of non-authoritative fact-checking significantly influenced participants’ cognitive processing, primarily by weakening the engagement of RPFC-mediated deliberative evaluation, leading to reduced cognitive conflict monitoring. In contrast, for false information, the addition of authoritative fact-checking emerged as a pivotal factor modulating the cognitive processes, suppressing the RPFC’s activation related to cognitive conflict detection, thereby shortening the evaluation process. Notably, behavioral data show that non-authoritative fact-checkers achieved higher accuracy (59%) than authoritative ones (55%) in judging false information. This finding contrasts with the neural data indicating an “authoritative source effect,” implying that authority does not always confer cognitive benefits. This may be attributed to non-authoritative sources, due to their lower perceived credibility, which fail to provide a reliable “fast track” for resolving cognitive conflict (Feng et al., 2023). Instead, they prompt participants to maintain higher vigilance or engage in more critical thinking, sustaining RPFC activation for longer periods to monitor and resolve potential discrepancies, thereby slightly enhancing the discrimination of false information at the behavioral level. This contrasting effect may be explained by cognitive dissonance theory (Festinger, 1962), wherein individuals confronted with incongruent or differentially credible external cues experience cognitive conflict and adjustment (McClellan et al., 2026). This alters their information processing strategies, which are closely linked to cognitive control and metacognitive monitoring (Moravec et al., 2019). Our findings are consistent with a recent study investigating cognitive conflict in processing misinformation (McClellan et al., 2026).

Furthermore, this study reinforces the intricate association between the brain’s cognitive control regions and information processing. Specifically, the left PFC exhibited significantly enhanced activation upon the introduction of authoritative fact-checking sources such as People’s Daily. The left PFC is activated in conflict monitoring, while conflict resolution engages bilateral prefrontal regions (Sun et al., 2013). This could be interpreted as authoritative information providing a clear and credible external cue that facilitates the resolution of initial cognitive conflict triggered by information evaluation (Moore et al., 2021). This effectively reduces the uncertainty in judgment and promotes more efficient integration of the information into existing knowledge frameworks, thereby enhancing the acceptance of accurate information. Conversely, the right PFC, typically associated with conflict monitoring and uncertainty assessment (Moravec et al., 2019; Sun et al., 2013), demonstrated specific patterns reflecting the cognitive challenges and sustained evaluation elicited by non-authoritative information. Reduced RPFC activation under non-authoritative fact-checking for true information reflects diminished conflict monitoring, while its relatively sustained activation under non-authoritative fact-checking for false information supports prolonged evaluation. Notably, the functional roles of these prefrontal subregions are closely tied to cognitive control processes. The LPFC is critically involved in cognitive control and working memory; in contrast, the RPFC plays a key role in conflict monitoring and uncertainty assessment, supporting the detection of discrepancies between information and prior knowledge. The observed activation patterns thus reflect a complex integration of source credibility appraisal and higher-order cognitive processing during information evaluation.

#### Changes in HbO concentrations

**Figure 4:**
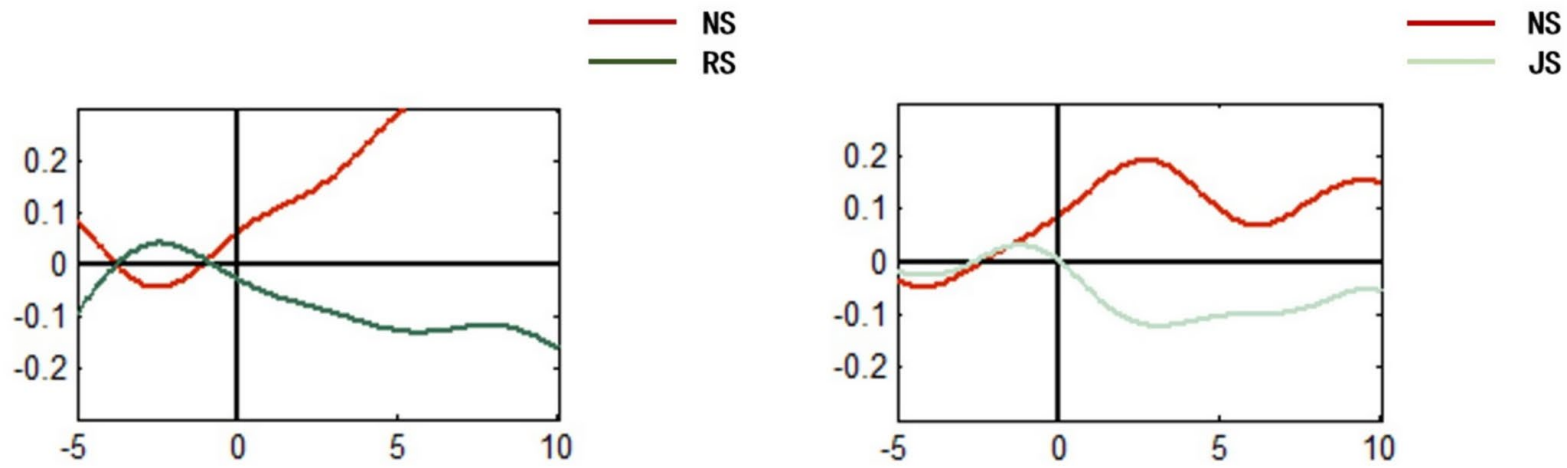
Grand averages of all subjects in the NS, RS, and JS conditions.

Figure 4 compares the changes in HbO within the specific PFC region over time (−5 to 10s), before and after the incorporation of third-party fact-checkers. The experimental data demonstrated the disparities between the two groups.

In the control condition for false information presented without third-party fact-checkers (NS condition, left panel), a rising trend in HbO concentration was observed, indicating increased activation in this brain region, likely reflecting engagement in conflict monitoring or elevated cognitive effect. This activation is likely engaged in monitoring information conflict or exerting cognitive effort to resolve uncertainties about the information’s veracity (Kawiak et al., 2020; McClellan et al., 2026). Conversely, in the experimental group where authoritative third-party fact-checkers were introduced (RS condition), a notable decline in HbO concentration was observed. This pronounced alteration suggests that the presence of authoritative fact-checking significantly impacts participants’ cognitive processes. Specifically, the high credibility of the source provides a strong external signal that resolves the initial cognitive conflict triggered by the false information (Feng et al., 2023), reducing the internal cognitive load for conflict resolution and uncertainty assessment. This reduction in cognitive demand manifests as attenuated neural activation in the PFC, which supports the hypothesis that authoritative sources can serve as a “fast track” in misleading contexts (Kahneman, 2012)—shortening the time required to resolve cognitive conflict but potentially limiting in-depth evaluation of the information itself.

Similar results were observed regarding true information, wherein the HbO in the specific RPFC region exhibits an upward trend, potentially reflecting neural activity related to baseline assessment of information authenticity or cognitive preparation, including the initial detection of consistency (or lack thereof) between the true information and prior knowledge, which may trigger low-level cognitive conflict that prompts further verification (McClellan et al., 2026). However, labeling authentic information with non-authoritative third-party fact-checkers altered the RPFC activation pattern. The trend in HbO concentration shows a downward trend, indicating reduced activation. This shift suggests that the introduction of a non-authoritative source may trigger a process of rapid trust calibration or source reliability evaluation. Participants may judge the non-authoritative source as less reliable, leading them to reduce their reliance on the source’s cues and, consequently, diminish the engagement of RPFC-mediated conflict monitoring and deliberative evaluation. This shift in neural pattern, consistent with the lack of significant improvement in behavioral accuracy, jointly points to the possibility that non-authoritative fact-checking may prompt a more efficiency-oriented, source-feature-based shallow processing strategy. The results showed that people reduce cognitive conflict-related activation without enhancing the accuracy of information judgment.

## Discussion

This study delves into the neural mechanisms underlying the impact of the credibility of third-party fact-checkers on individuals’ cognitive processing, utilizing fNIRS. These findings offer a novel perspective and empirical evidence for understanding information recipients’ decision-making behaviors and enhancing their information discrimination abilities. The experimental results not only confirm the pivotal role of authoritative third-party fact-checkers in true information processing but also reveal a unique phenomenon where both authoritative and non-authoritative fact-checking significantly enhance judgment accuracy in the context of false information.

Firstly, in the processing of true information, the introduction of authoritative third-party fact-checkers (e.g., People’s Daily) markedly elevates participants’ judgment accuracy, yielding an increase from a baseline of 37 to 62%. This outcome reflects previous research, further emphasizing the irreplaceable nature of authoritative fact-checking in shaping users’ cognitive frameworks and information processing strategies (Xu et al., 2023). The fNIRS data provide neural-level evidence, indicating that the incorporation of authoritative fact-checking activates the left prefrontal cortex (left PFC), a neural substrate for cognitive conflict resolution and working memory integration (McClellan et al., 2026; Sun et al., 2013). This significant enhancement may suggest that authoritative information provides a reliable external cue that effectively resolves initial cognitive conflict triggered by information evaluation, thereby facilitating efficient integration of the information into existing knowledge structures and promoting accurate judgment. This process encompasses not only psychological mechanisms of trust transfer but also the influence of cognitive biases, which reduces the cognitive load required for verification and enables users to readily accept and rely on accurate information (Feng et al., 2023; Moore et al., 2021). Additionally, a notable change in the activation pattern of the right PFC (Miller and Cohen, 2001) was observed when participants were presented with true information accompanied by non-authoritative fact-checking. Specifically, exposure to true information only elicited the activation of the RPFC, possibly reflecting the brain’s initial skepticism or evaluation of information authenticity. However, when true information was presented with non-authoritative fact-checking, the activation pattern of the right PFC underwent a shift: activation intensity decreased significantly. Combined with the behavioral data showing only a marginal and non-significant increase in accuracy, this alteration in neural activity likely does not reflect an enhancement of a “deliberative assessment mechanism.” Instead, it more plausibly indicates that participants reduced their cognitive investment in the information or adopted a heuristic processing strategy based on the perceived low credibility of the source (Moravec et al., 2019), engaging in rapid “cognitive offloading” that diminishes the engagement of RPFC-mediated cognitive conflict monitoring and detailed evaluation.

Contrasting with true information processing, this study uncovers an intriguing phenomenon in the handling of false information. Both authoritative and non-authoritative third-party fact-checkers significantly enhanced participants’ accuracy in judging false information, leading to an increase from 36 to 55 and 59%, respectively. Notably, behavioral data showed that non-authoritative fact-checkers achieved higher accuracy (59%) than authoritative ones (55%) in judging false information. This finding contrasts with the neural data indicating an “authoritative source effect,” implying that authority does not always confer cognitive benefits in resolving cognitive conflict (Metzger et al., 2020). This may be attributed to non-authoritative sources, due to their lower perceived credibility, which triggered a “fast track” for resolving cognitive conflict (Moore et al., 2021). Instead, authoritative sources prompt participants to maintain higher vigilance or engage in more critical thinking, sustaining RPFC activation to monitor discrepancies between the false information and prior knowledge (McClellan et al., 2026), thereby enhancing the detection of inaccuracies. These results suggest that authoritative fact-checking often accelerates cognitive conflict resolution in the dissemination of false information, while non-authoritative fact-checking prolongs conflict monitoring to ensure more thorough verification. Analysis of fNIRS data revealed that exposure to false information elicited significantly different activation patterns of the right PFC when labeled with different third-party fact-checkers. Specifically, exposure to false information only was found to consistently activate the RPFC, possibly reflecting the brain’s initial skepticism or evaluation of information authenticity, which triggers cognitive conflict signals that prompt further assessment (Festinger, 1962). However, integration of false information with authoritative fact-checking resulted in a significant decrease in the activation state of the RPFC in specific channels, strongly suggesting that authoritative fact-checking may serve as a “fast track” in misleading contexts (Kahneman, 2012). This “fast track” effect manifests as participants using the source’s high credibility to quickly resolve initial cognitive conflict (Feng et al., 2023), thereby engaging in less intensive cognitive deliberation and assessment of the information itself, which accelerates decision-making but may reduce the depth of conflict monitoring (Moravec et al., 2019; Kahneman, 2012).

This contrasting pattern can be explained by cognitive dissonance theory (Metzger et al., 2020), whereby incongruent or differentially credible external cues experience cognitive conflict and adjustment, thereby dynamically altering their information processing strategies (McClellan et al., 2026). Specifically, authoritative sources reduce cognitive dissonance by providing reliable cues (Moore et al., 2021), while non-authoritative sources either fail to resolve dissonance (for false information) or diminish the need to address it (for true information). Furthermore, the accuracy values indicated that in the absence of third-party fact-checkers, the accuracy rates for both true and false information were 37 and 36%. These results are consistent with those of Moravec et al., stating that dominance of confirmation bias substantially impairs users’ ability to discriminate true and false information (Moravec et al., 2019). This impairment likely stems from weakened cognitive conflict detection: individuals prioritize information that aligns with prior beliefs, reducing the activation of PFC regions responsible for monitoring discrepancies (McClellan et al., 2026). This leads to unreliable assessments regarding the veracity of the information presented.

Moreover, this study further validates the intricate connection between the brain’s cognitive control regions and information processing. The left PFC showed significantly enhanced activation under authoritative fact-checking conditions, reflecting the facilitative role in cognitive conflict resolution and working memory integration (Miller and Cohen, 2001). Conversely, the decreased activation of the right PFC under exposure to non-authoritative fact-checking for true information may stem from its involvement in conflict monitoring and uncertainty assessment (Moravec et al., 2019). Sources perceived as unreliable result in reduced investment in conflict detection, leading to diminished activation. This discovery highlights the complex interplay between cognitive conflict modulation and deliberative evaluation during information processing.

Consequently, the following recommendations are proposed. In information dissemination, the authority and transparency of third-party fact-checkers should be prioritized to provide reliable cues for cognitive conflict resolution (Feng et al., 2023), ensuring information reliability while avoiding blind reliance on authoritative sources. Simultaneously, users should actively screen and verify information, fostering critical thinking skills that enhance cognitive conflict detection. This implies sustained engagement of PFC regions to monitor discrepancies between information and prior knowledge (McClellan et al., 2026). Furthermore, cognitive conflict mechanisms should be leveraged to optimize information dissemination strategies, such as designing interventions that prompt appropriate levels of conflict monitoring. This approach represents a crucial direction for future information communication research.

In conclusion, the present study utilized fNIRS to unveil the profound influence of the credibility of third-party fact-checkers on the cognitive processing mechanisms. These findings provide a pivotal theoretical and practical foundation for optimizing information dissemination strategies and enhancing individuals’ information discrimination capabilities. Future research endeavors should delve deeper into the interplay of diverse information types, individual characteristics, and contextual factors in shaping the cognitive processing dynamics, thereby developing a more comprehensive and nuanced model of information processing. Additionally, an in-depth exploration of the role of cognitive conflict in the dissemination of information would facilitate a more effective harnessing of this mechanism. This would increase public trust in information and enhance discrimination abilities by promoting appropriate conflict monitoring and resolution, ultimately contributing to the establishment of a healthy and orderly information ecosystem.

## Conclusion

This study operationalized the credibility of third-party fact-checkers based on institutional authority, derived from an organization’s official status and public credibility. This study selected People’s Daily (a high-authority, central-level official outlet) and Jiupai News (a lower-authority, local-market platform) as contrasting sources, a choice anchored in established credibility research documenting a “stronger central, weaker local” media landscape. The validity of this operational definition is further substantiated by the behavioral results of this study. A chi-square test analysis of judgment accuracy for true information revealed a significant difference in the distribution of accuracy across the different source conditions (no source, Jiupai News, People’s Daily; χ^2^ (2, *N* = 264) = 11.092, *p* = 0.004). Specifically, when information was tagged with People’s Daily, participants’ accuracy in judging it as “true” was highest (62%). When information was tagged with Jiupai News, judgment accuracy was intermediate (45%). In the absence of any source labeling, judgment accuracy was lowest (37%). The judgment accuracy of information labeled with People’s Daily was higher than that of Jiupai News. This finding supports the validity of the manipulation of “institutional authority” variable, confirming differences in cognitive processing.

This study focuses on the profound influence of the credibility of third-party fact-checkers on cognitive processing. Functional near-infrared spectroscopy (fNIRS) was performed to investigate the differences in neural activity when processing information verified by authoritative versus non-authoritative third-party fact-checkers. To examine whether experimental repetitions induced practice or fatigue effects that could confound the core findings, a judgment stability analysis was conducted for all 16 information stimuli. For each piece of information, Cochran’s Q tests were performed to assess whether the distribution of participants’ judgments (true/false) remained consistent across the five repeated presentations. The statistical analysis results indicated that none of the tests for the 16 pieces of information reached statistical significance (all *p* > 0.05). This demonstrates that participants’ judgments for each specific piece of information remained stable across the five repetitions, with no systematic change. Consequently, the observed between-condition differences in behavioral and neural patterns in this experiment can be robustly attributed to the experimental manipulation (i.e., source authority), rather than to temporal effects arising from repeated presentation.

The findings indicate that authoritative third-party fact-checkers exhibit significant advantages in enhancing the evaluation accuracy of true information; this is evidenced by increased activation in the left prefrontal cortex, reflecting the pivotal roles of trust transfer and cognitive conflict resolution in information processing. Regarding true information, the introduction of non-authoritative fact-checking was associated with a decrease in RPFC activation. This reduction, coupled with only a marginal improvement in behavioral accuracy, suggests a shift toward heuristic processing or cognitive offloading rather than enhanced deliberative assessment. When confronted with false information, authoritative third-party fact-checkers serve as a “fast track,” urging participants to reduce in-depth contemplation and evaluation of the information, thereby improving judgment accuracy to a certain extent but potentially overlooking the need for thorough verification of the information’s authenticity. Notably, behavioral data revealed that judgment accuracy for false information was slightly higher when accompanied by non-authoritative fact-checkers (59%) compared to authoritative ones (55%). This conflicts with the neural “fast track,” suggesting that non-authoritative sources may, in some contexts, inadvertently promote more vigilant assessment. Consequently, RPFC activation is sustained, indicating active cognitive conflict monitoring, which enhances the detection of false information. Meanwhile, non-authoritative third-party fact-checking also demonstrates the potential to promote cautious assessment, challenging conventional cognitions about source authority and emphasizing the complexity and multifaceted nature of information processing. This pattern of results, where source authority differentially modulates processing based on information veracity, can be interpreted through the lens of cognitive dissonance theory (Chandra and Choudhury, 2023), as individuals adjust their processing strategies in response to incongruent cues.

This research not only deepens our understanding of the relationship between information credibility and cognitive processing but also provides crucial empirical evidence for optimizing information dissemination strategies. In the information era, the authority of third-party fact-checkers can be utilized to enhance public information discrimination capabilities, promoting careful consideration of information content authenticity, individual cognitive characteristics, and contextual factors. While authority can enhance efficiency and trust in some scenarios, it may also shorten active content evaluation, whereas non-authoritative sources might, under specific conditions, elicit additional scrutiny. Furthermore, the application of fNIRS technology opens up new avenues for cognitive science research, demonstrating its unique value in uncovering the intricacies of the brain’s cognitive processes.

In summary, this study adopts an interdisciplinary methodology integrating perspectives from psychology, cognitive science, and neuroscience, and contributes novel insights into understanding information receivers’ psychological mechanisms. The findings facilitate the optimization of the information dissemination environment and promote the construction of a healthy information ecosystem. Future research should focus on exploring the impacts of different information types, individual differences, and socio-cultural environments on cognitive processing.

## Data Availability

The original contributions presented in the study are included in the article/supplementary material, further inquiries can be directed to the corresponding authors.
